# EGFR signaling promotes inflammation and cancer stem-like activity in inflammatory breast cancer

**DOI:** 10.18632/oncotarget.18958

**Published:** 2017-07-04

**Authors:** Xiaoping Wang, Monica E. Reyes, Dongwei Zhang, Yohei Funakoshi, Adriana P. Trape, Yun Gong, Takahiro Kogawa, Bedrich L. Eckhardt, Hiroko Masuda, David A. Pirman, Peiying Yang, James M. Reuben, Wendy A. Woodward, Chandra Bartholomeusz, Gabriel N. Hortobagyi, Debu Tripathy, Naoto T. Ueno

**Affiliations:** ^1^ Morgan Welch Inflammatory Breast Cancer Research Program and Clinic, The University of Texas MD Anderson Cancer Center, Houston, Texas, USA; ^2^ Section of Translational Breast Cancer Research, The University of Texas MD Anderson Cancer Center, Houston, Texas, USA; ^3^ Department of Breast Medical Oncology, The University of Texas MD Anderson Cancer Center, Houston, Texas, USA; ^4^ Department of Pathology, The University of Texas MD Anderson Cancer Center, Houston, Texas, USA; ^5^ Department of Cancer Biology, The University of Texas MD Anderson Cancer Center, Houston, Texas, USA; ^6^ Department of General Oncology, The University of Texas MD Anderson Cancer Center, Houston, Texas, USA; ^7^ Department of Hematopathology, The University of Texas MD Anderson Cancer Center, Houston, Texas, USA; ^8^ Department of Radiation Oncology, The University of Texas MD Anderson Cancer Center, Houston, Texas, USA

**Keywords:** inflammatory breast cancer, EGFR, COX-2, nodal, cancer stem-like cells

## Abstract

Inflammatory breast cancer (IBC) is the most lethal and aggressive type of breast cancer, with a strong proclivity to metastasize, and IBC-specific targeted therapies have not yet been developed. Epidermal growth factor receptor (EGFR) has emerged as an important therapeutic target in IBC. However, the mechanism behind the therapeutic effect of EGFR targeted therapy is not well defined. Here, we report that EGFR regulates the IBC cell population that expresses cancer stem-like cell (CSC) markers through COX-2, a key mediator of inflammation whose expression correlates with worse outcome in IBC. The COX-2 pathway promoted IBC cell migration and invasion and the CSC marker-bearing population *in vitro*, and the inhibition of this pathway reduced IBC tumor growth *in vivo*. Mechanistically, we identified Nodal, a member of the TGFβ superfamily, as a potential driver of COX-2-regulated invasive capacity and the CSC phenotype of IBC cells. Our data indicate that the EGFR pathway regulates the expression of COX-2, which in turn regulates the expression of Nodal and the activation of Nodal signaling. Together, our findings demonstrate a novel connection between the EGFR/COX-2/Nodal signaling axis and CSC regulation in IBC, which has potential implications for new combination approaches with EGFR targeted therapy for patients with IBC.

## INTRODUCTION

Inflammatory breast cancer (IBC) is the most lethal and aggressive form of breast cancer; it accounts for 2–4% of breast cancer cases but causes 8–10% of breast cancer-related deaths in the United States [1, 2]. IBC tumors have features associated with poor prognosis, such as overexpression of HER2, EGFR, E-cadherin, and nuclear factor κB [3]. IBC is also associated with a high rate of distant metastasis [4, 5]. To date, there are no FDA-approved targeted therapies that are specific for IBC. Several molecular changes have been identified in IBC, including loss of WNT1-inducible signaling pathway protein-3 (WISP3) and overexpression of Rho GTPase [6], E-cadherin [7], angiogenic factors [8], translation initiation factor eIF4GI [9], and tazarotene-induced gene 1 (TIG1) [10]. However, the molecular mechanism underlying aggressiveness of IBC is not well understood. One proposed mechanism contributing to the aggressiveness of IBC is enrichment for cancer stem-like cells (CSCs). It has been shown that the metastatic, aggressive behavior of IBC is mediated by a CSC component that displays aldehyde dehydrogenase 1 (ALDH1) enzymatic activity [11]. Thus, development of novel effective therapies targeting CSCs in IBC may significantly improve outcomes of patients with IBC.

Epidermal growth factor receptor (EGFR) is overexpressed in all subtypes of breast cancer, particularly in IBC and triple-negative breast cancer [12–15]. EGFR expression is an independent prognostic marker for a high rate of recurrence and shorter survival in IBC patients [12]. Our recently completed single-arm phase II study of panitumumab (PmAb), a human IgG2 anti-EGFR monoclonal antibody, combined with chemotherapy (NCT01036087) showed the highest ever observed pathological complete tumor response (pCR) rate to preoperative treatment in patients with HER2-negative IBC (29%, compared to a historical pCR rate of 15%); of note, a pCR rate of 47% was observed in the triple-negative subset [16]. In our previous preclinical study, EGFR tyrosine kinase inhibition changed the phenotype of IBC cells from mesenchymal to epithelial and inhibited IBC tumor growth and metastasis [15]. These findings indicate that EGFR is an important therapeutic target in IBC. It is critical to define the mechanism behind this therapeutic effect and develop effective combination therapies to augment the effects of anti-EGFR therapy for patients with IBC.

A large body of evidence indicates that chronic inflammation may contribute to a variety of cancers, including breast cancer [16, 17]. Cyclooxygenase-2 (COX-2) is an inducible isozyme that catalyzes the conversion of arachidonic acid, a key inflammatory intermediate, to prostaglandins and other prostanoids, which are involved in various aspects of the inflammatory response. COX-2 is elevated in a number of malignancies, and its overexpression is associated with increased cancer cell growth, increased invasiveness, and a poor prognosis in patients with breast cancer [18–21]. A selective COX-2 inhibitor, celecoxib, has demonstrated preclinical and clinical efficacy in reducing the risk of breast cancer development [22]. IBC is so named because of its characteristic “inflammatory-like” clinicopathological manifestations such as diffuse erythema and edema of the breast. However, the molecular mechanism of how inflammation contributes to the aggressiveness of IBC remains elusive, and whether there is a functional link between EGFR and COX-2 in IBC has not been determined.

Given that CSCs may be a critical contributor to the aggressiveness of IBC and the inflammatory-like clinicopathological characteristic of IBC, we herein investigated the role of the EGFR pathway in the regulation of IBC CSCs and COX-2. Our findings demonstrate a novel signaling axis among EGFR, COX-2, and Nodal (a member of the TGFβ superfamily) that regulates the enrichment of the CSC population in IBC. These findings may lead to the development of combinatorial approaches with EGFR targeted therapy for patients with IBC.

## RESULTS

### Inhibition of the EGFR pathway impairs IBC stemness

**Figure 1:**
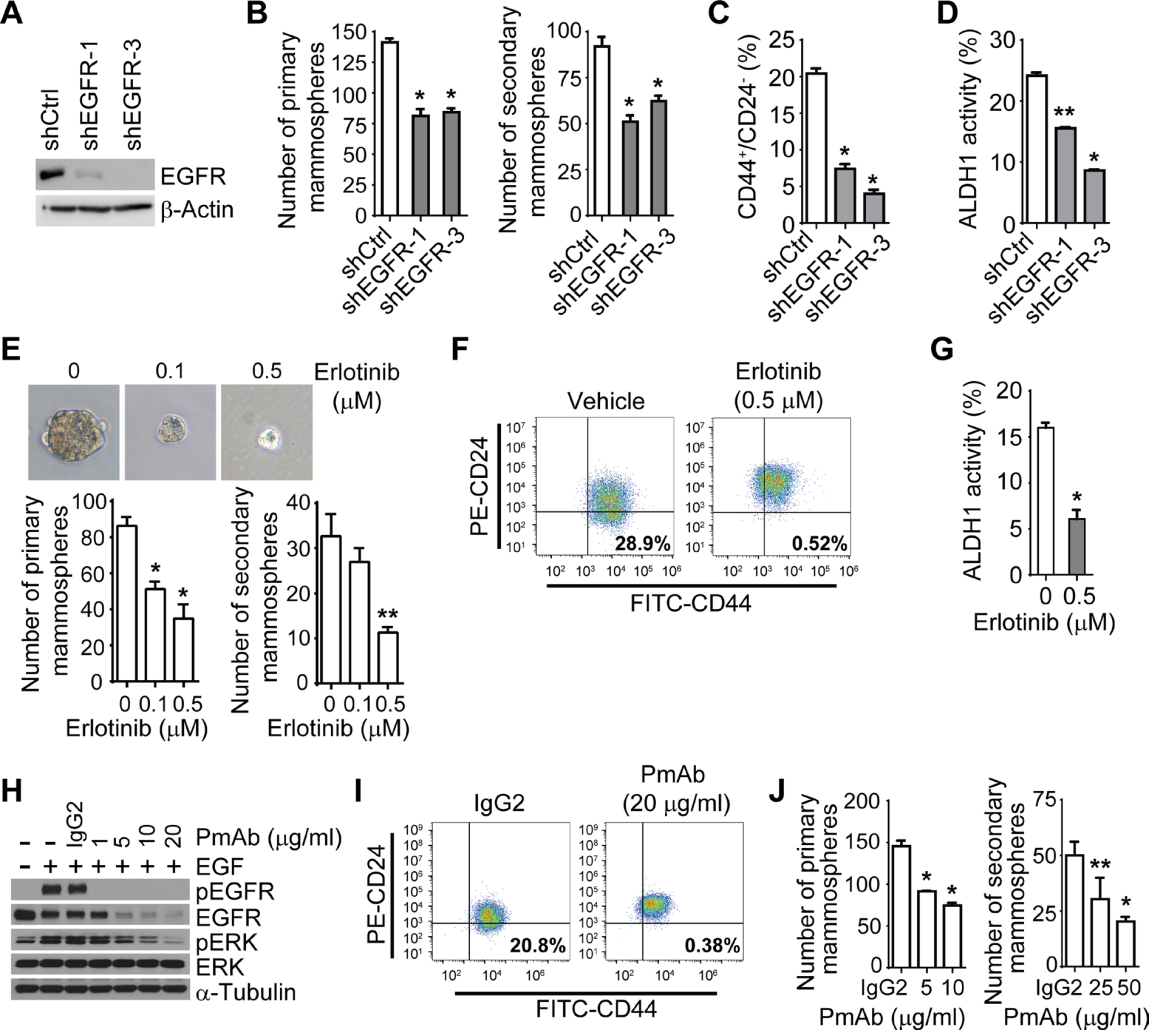
The EGFR pathway regulates the IBC cell population that expresses CSC markers (**A**) EGFR expression is depleted in SUM149 cells after EGFR knockdown. Expression of EGFR in the stable shCtrl clone and EGFR knockdown clones, shEGFR-1 and shEGFR-3, was analyzed by Western blotting. (**B**) EGFR depletion reduces the formation of mammospheres in SUM149 cells. Bars, ± SD. *P < 0.001. (**C** and **D**). EGFR depletion decreases the CD44^+^/CD24^−/low^ population (C) and ALDH activity (D) of SUM149 cells. *P < 0.001; **P < 0.01. (**E**) Erlotinib treatment decreases mammosphere formation of SUM149 cells. Shown are representative images of primary mammospheres. *P < 0.001; **P = 0.001. (**F**) and (**G**) Erlotinib treatment decreases the CD44^+^/CD24^−/low^ population (F) and ALDH activity (G) of SUM149 cells. FITC, fluorescein isothiocyanate; PE, phycoerythrin. *P < 0.01. (**H**) Panitumumab (PmAb) treatment inhibits EGF-stimulated phosphorylation of EGFR in SUM149 cells. Cells were pretreated with PmAb at the indicated doses for 1 hour and then stimulated with EGF (50 ng/mL) for 15 minutes. (**I**) PmAb treatment decreases the CD44^+^/CD24^−/low^ population of SUM149 cells. (**J**) PmAb treatment decreases the mammosphere formation of SUM149 cells. *P < 0.001; **P < 0.005. Experiments were independently repeated 3 times.

To understand the role of EGFR in regulating the aggressiveness of IBC, we assessed the impact of the EGFR pathway on the IBC cell population expressing CSC markers, defined as the CD44^+^/CD24^−/low^ or ALDH^+^ fraction, and on mammosphere formation. We first knocked down the expression of EGFR in IBC SUM149 cells (Figure 1A) and found that compared with the stable control clone, the EGFR-depleted shEGFR-1 and shEGFR-3 clones formed fewer primary and secondary mammospheres and had lower CD44^+^/CD24^−/low^ and ALDH^+^ subpopulations (Figure 1B–1D and Supplementary Figure 1A, 1B). Inactivation of EGFR signaling by erlotinib treatment decreased the formation of mammospheres and the CD44^+^/CD24^−/low^ and ALDH^+^ subpopulations in SUM149 cells (Figure 1E–1G and Supplementary Figure 1C). Similar results were observed in IBC KPL-4 and MDA-IBC3 cells (Supplementary Figure 2A–2C).

Because EGFR tyrosine kinase inhibitors may have a non-specific effect due to suppressing other kinases, we next treated IBC cells with panitumumab (PmAb), a human IgG2 anti-EGFR monoclonal antibody. PmAb treatment inactivated the EGFR pathway in SUM149 cells, as indicated by the abolishment of the phosphorylation of EGFR and downstream molecule ERK upon EGF stimulation (Figure 1H). As shown in Figure 1I and 1J, PmAb treatment decreased the CD44^+^/CD24^−/low^ fraction and mammosphere formation in SUM149 cells. Similar results were observed in FC-IBC-02 and MDA-IBC3 cells (Supplementary Figure 2D and 2E). Taken together, these results indicate that the EGFR pathway regulates the CSC marker-bearing population in IBC cells.

### The COX-2 inflammatory pathway is functionally linked to EGFR signaling in IBC cells

**Figure 2:**
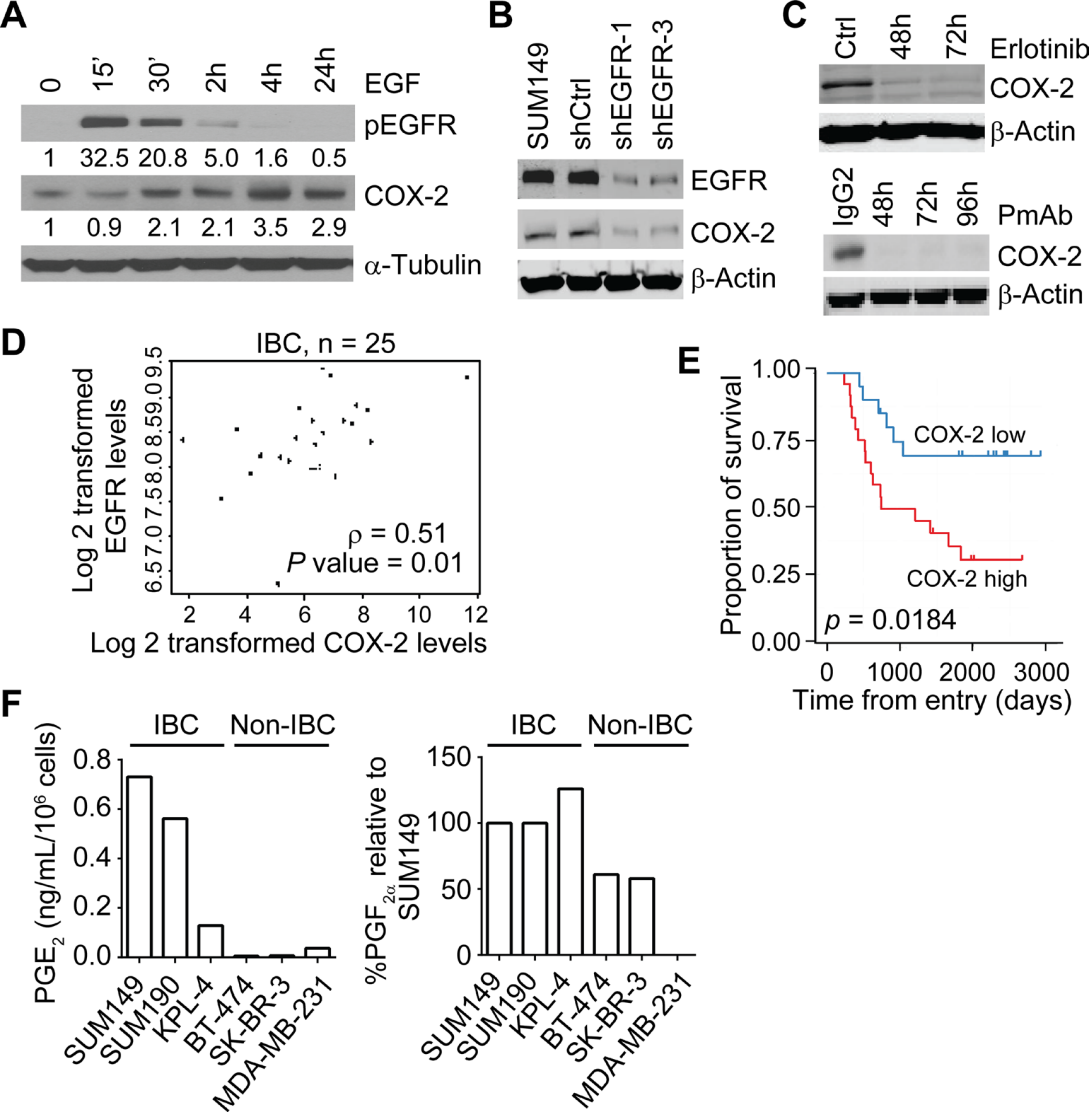
The EGFR pathway regulates COX-2 in IBC (**A**) EGF stimulation increases the expression of COX-2 in SUM149 cells. Cells were serum-starved for 24 hours prior to stimulation with recombinant EGF at 100 ng/mL for the indicated time periods. Ctrl, control. (**B**) EGFR knockdown reduces the expression of COX-2 in SUM149 cells. (**C**) Erlotinib or PmAb treatment reduces the expression of COX-2 in SUM149 cells. (**D**) EGFR and COX-2 mRNA expression correlates in 25 IBC patient biopsy samples. (**E**) In a different population of 44 IBC patients, high COX-2 expression in tumor specimens correlates with worse overall survival. *P* = 0.0184. (**F**) IBC cells have higher levels of COX-2's enzymatic products, PGE_2_ and PGF_2α_, than non-IBC cells. Experiments in panels A, B, and C were independently repeated 3 times.

To determine the role of the EGFR pathway in inflammation of IBC, we first assessed the regulation of COX-2, a key molecule in the inflammatory response, by EGFR signaling. As shown in Figure 2A and 2B, the COX-2 expression level was upregulated in SUM149 cells following EGF stimulation (Figure 2A) and notably downregulated in SUM149 cells stably transfected with EGFR shRNA (Figure 2B). COX-2 expression decreased when the EGFR pathway in SUM149 cells was inactivated by erlotinib or PmAb treatment (Figure 2C). Similar results were obtained in SUM190, KPL-4, and FC-IBC-02 cells (Supplementary Figure 3A and 3B). We then used cDNA microarray gene expression data to examine the mRNA expression levels of COX-2 (204748_at)and EGFR (211551_at) in tumor biopsy specimens from 25 patients with IBC. We found that the expression of COX-2 significantly correlated with the expression of EGFR in these specimens (*P* = 0.01) (Figure 2D). These results suggest that EGFR functionally regulates COX-2 activity in IBC.

We further assessed the clinical relevance of COX-2 in IBC by performing immunohistochemical staining of COX-2 in tissues from a different group of 44 patients with primary IBC and found that patients with high COX-2 expression had significantly worse overall survival than those with low COX-2 expression (Figure 2E). High expression of COX-2 was correlated with higher nuclear grade of IBC tumors (Supplementary Table 1). We also found that IBC cell lines had higher levels of COX-2′s enzymatic products, PGE_2_ and PGF_2α_, than did noninflammatory breast cancer (non-IBC) cell lines as measured by HPLC-MS/MS (Figure 2F). Taken together, these results highlight the significance of COX-2 in the progression of IBC and warrant further investigation of the contribution of EGFR/COX-2 to IBC aggressiveness.

### COX-2 mediates the EGFR-regulated CSC phenotype in IBC cells

**Figure 3:**
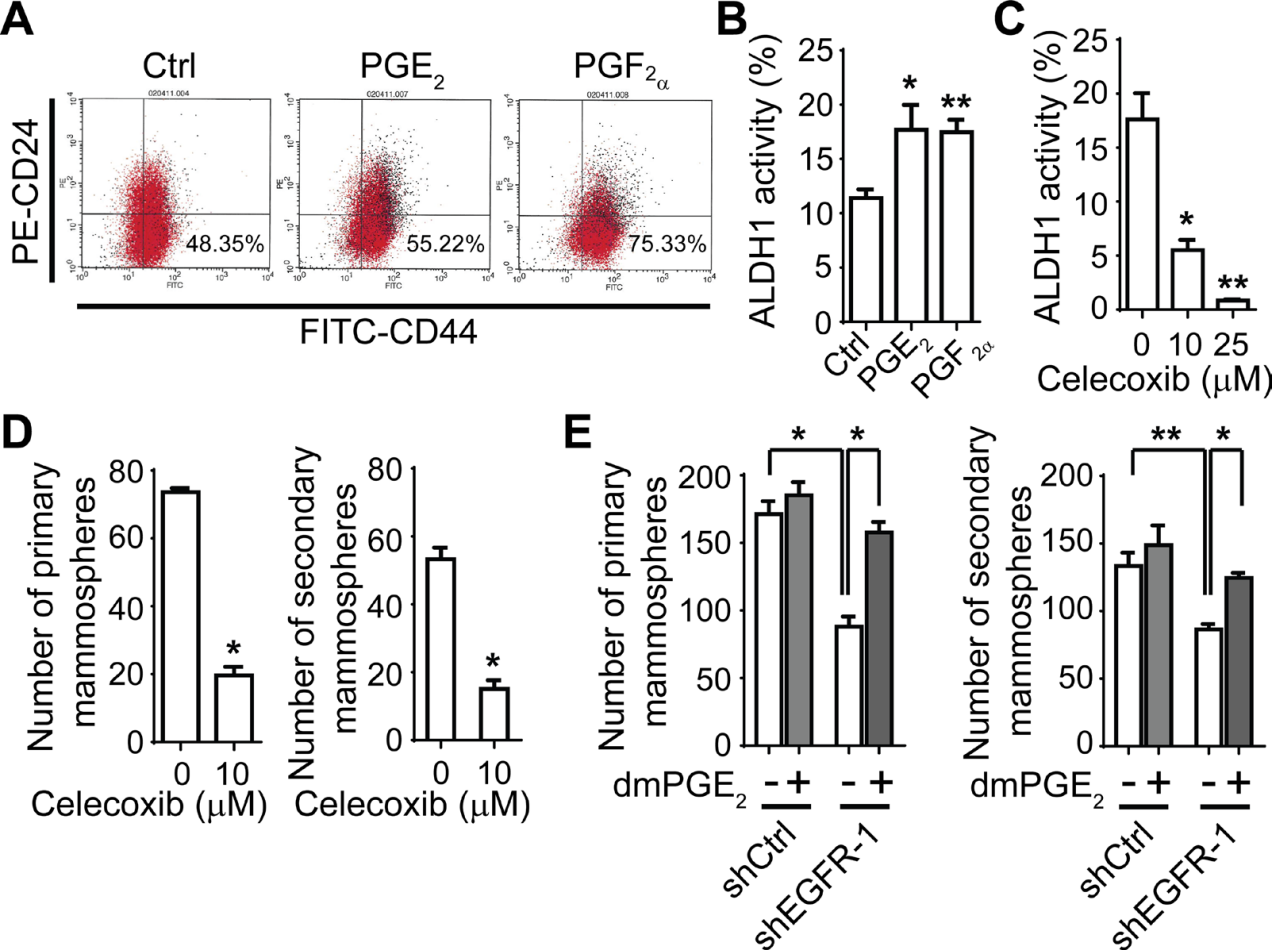
The COX-2 pathway regulates the IBC cell population that expresses CSC markers (**A**) and (**B**) PGE_2_ and PGF_2α_ treatment increases the CD44^+^/CD24^−/low^ population (A) and ALDH activity (B) of SUM149 cells. Cells were treated with 0.5 μM PGE_2_ or PGF_2α_ for 48 hours and then subjected to flow cytometry analysis. *P = 0.01; **P = 0.001. (**C**) Treatment with COX-2 inhibitor celecoxib decreases ALDH activity of SUM149 cells. Cells were treated with celecoxib at the indicated doses for 48 hours and then subjected to flow cytometry analysis. *P = 0.001; **P < 0.001. (**D**) Celecoxib treatment decreases mammosphere formation of SUM149 cells. *P < 0.005. (**E**) A stabilized PGE_2_ analogue, dmPGE_2_ (100 nM), mitigates the decrease of primary (left) and secondary (right) mammosphere formation in SUM149 EGFR-knockdown clone shEGFR-1. *P < 0.005; **P = 0.01. Experiments were independently repeated 3 times.

We next asked whether COX-2 is involved in the EGFR-regulated CSC phenotype in IBC cells. To address this question, we first studied the role of COX-2 in the regulation of the CSC phenotype. We treated SUM149 cells with PGE_2_ and PGF_2α_ and found that these treatments increased the subpopulation of CD44^+^/CD24^−/low^ and ALDH activity (Figure 3A and 3B), suggesting that prostaglandins promote CSC progenitors in IBC. Treatment with celecoxib, a COX-2 inhibitor, significantly inhibited the ALDH activity of SUM149 cells (Figure 3C) and reduced the formation of SUM149 (Figure 3D) and KPL-4 (Supplementary Figure 4A) mammospheres. These results imply that targeting COX-2 can inhibit the IBC cell population that expresses CSC markers. To further evaluate the role of COX-2 in the EGFR-regulated CSC phenotype in IBC, we added exogenous dimethyl PGE_2_ (dmPGE_2_), a stabilized PGE_2_ analogue, into the mammosphere culture of an EGFR-depleted clone. As shown in Figure 3E, the addition of dmPGE_2_ mitigated the inhibitory effect of EGFR knockdown on primary and secondary mammosphere formation of SUM149 cells. These results suggest that the EGFR-regulated CSC marker-bearing population in IBC is mediated by COX-2.

### COX-2 promotes an EMT-like phenotype, invasion, and tumor growth of IBC cells

**Figure 4:**
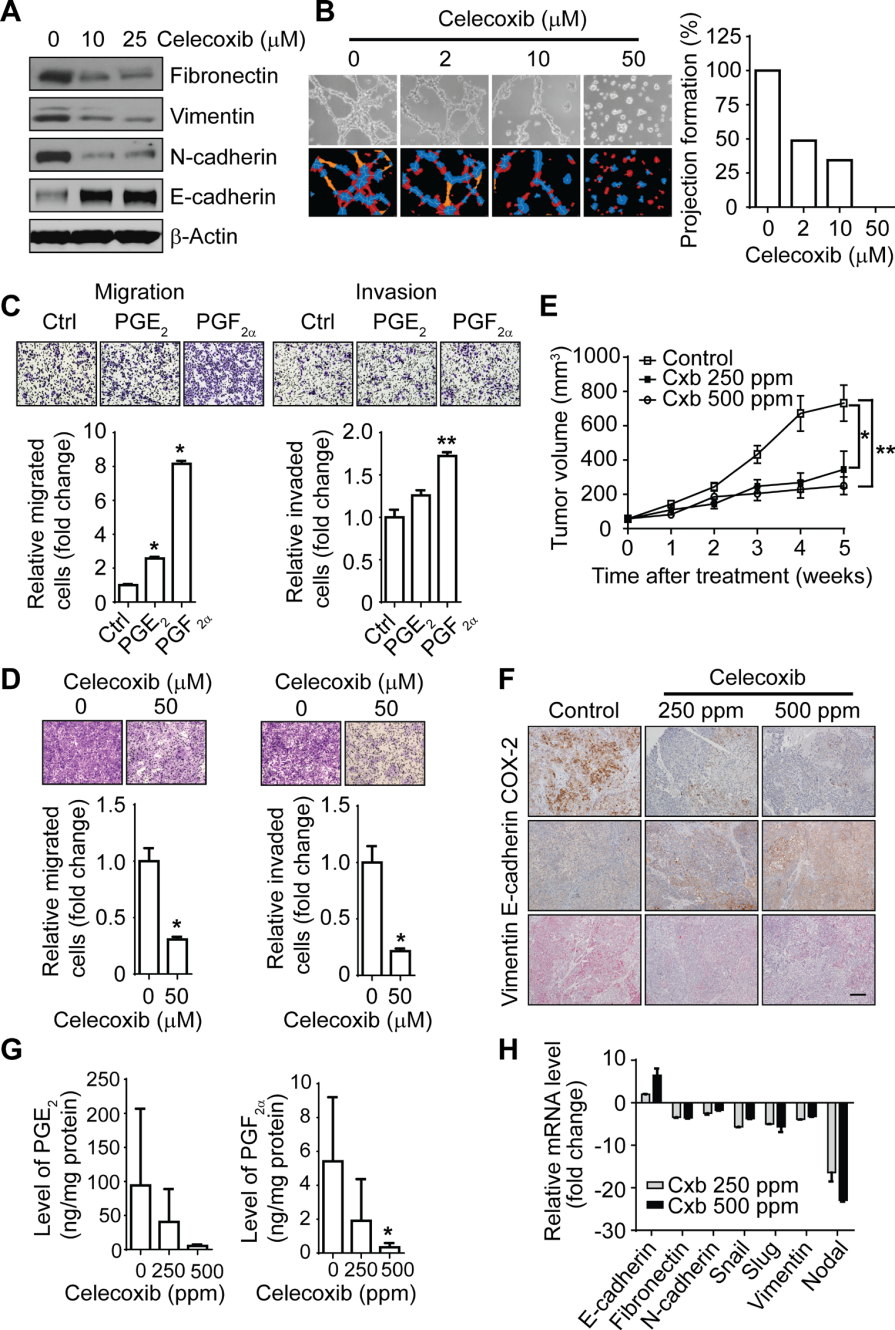
The COX-2 pathway regulates the EMT-like phenotype and invasiveness of IBC cells *in vitro* and tumor growth *in vivo* (**A**) Celecoxib treatment reduces the mesenchymal phenotype in SUM149 cells. (**B**) Celecoxib treatment reduces projection formation of SUM149 cells. Cells were plated in Matrigel culture with or without celecoxib for 48 hours. Left panel: Representative images of SUM149 cells. Right panel: Projections quantitated by S.CORE analysis. (**C**) PGE_2_ (0.5 μM) and PGF_2α_ (0.5 μM) treatment increases the migration (left panel) and invasion (right panel) of SUM149 cells. *P < 0.001; **P < 0.005. (**D**) Celecoxib treatment at the indicated dose for 48 hours decreases the migration (left panel) and invasion (right panel) of SUM149 cells. *P < 0.01. (**E**) Celecoxib treatment inhibits tumor growth in a SUM149 xenograft model. Each data point represents the mean tumor volume of eight mice per group. Cxb, celecoxib. Bars, SD. *P = 0.02; **P = 0.001. (**F**) Celecoxib treatment affects the expression of COX-2, E-cadherin, and vimentin in tumor tissues of SUM149 xenografts. Scale bar, 100 μm. (**G**) Celecoxib treatment reduces the expression of PGE_2_ and PGF_2α_ in SUM149 xenografts. *P = 0.01. (**H**) Celecoxib treatment affects the mesenchymal phenotype in SUM149 xenografts. Experiments in panels A, B, C, D, G, and H were independently repeated 3 times.

We further studied the role of COX-2 in IBC migration, invasion, and tumor growth. As shown in Figure 4A, treatment with celecoxib reduced the expression of mesenchymal markers fibronectin, vimentin, and N-cadherin and increased the expression of epithelial marker E-cadherin. Treating 3D cultures of SUM149 and KPL-4 cells with incremental doses of celecoxib blocked their invasive capacity, as evidenced by a reduction in cellular projections (Figure 4B and Supplementary Figure 4B). PGE_2_ and PGF_2α_ induced migration and invasion of SUM149 (Figure 4C) and invasion of KPL-4 (Supplementary Figure 4C) cells. This phenotype was functionally linked to the COX-2 pathway, as treatment with celecoxib reduced migration and invasion of SUM149 (Figure 4D) and KPL-4 (Supplementary Figure 4D) cells.

We further evaluated the clinical application of targeting COX-2 in IBC by investigating the efficacy of celecoxib in inhibiting IBC tumor growth and EMT *in vivo*. We implanted SUM149 tumor xenografts into the mammary fat pads of athymic *nu/nu* mice. Starting 3 weeks after implantation, mice with established tumors were administered celecoxib for 5 weeks. At both doses of celecoxib, 250 ppm and 500 ppm, we observed a significant mean inhibition of tumor growth: 57.3% in the 250-ppm group (*P* = 0.02 *P* = 0.0215) and 71.5% in the 500-ppm group (*P* = 0.001 *P* = 0.0011) compared to controls (Figure 4E). These doses of celecoxib did not produce toxicity issues *in vivo*. As expected, the expression levels of COX-2, PGE_2_, and PGF_2a_ were significantly reduced in the primary tumors of mice administered celecoxib (Figure 4F and 4G). The primary tumors of mice treated with celecoxib had increased expression of the epithelial marker E-cadherin and reduced expression of the mesenchymal markers fibronectin, N-cadherin, Snail, Slug, and vimentin at the mRNA level (Figure 4H). High expression of E-cadherin and low expression of vimentin at the protein level were detected by immunohistochemistry in celecoxib-treated tumors compared with untreated tumors (Figure 4F). These data demonstrate that COX-2 inhibition reverses the EMT phenotype and suppresses IBC tumor growth *in vivo*. Taken together, our findings indicate that the COX-2 pathway plays critical roles in invasion and tumor growth of IBC.

### EGFR/COX-2 signaling regulates the CSC phenotype and invasiveness of IBC cells via modulation of Nodal signaling

**Figure 5:**
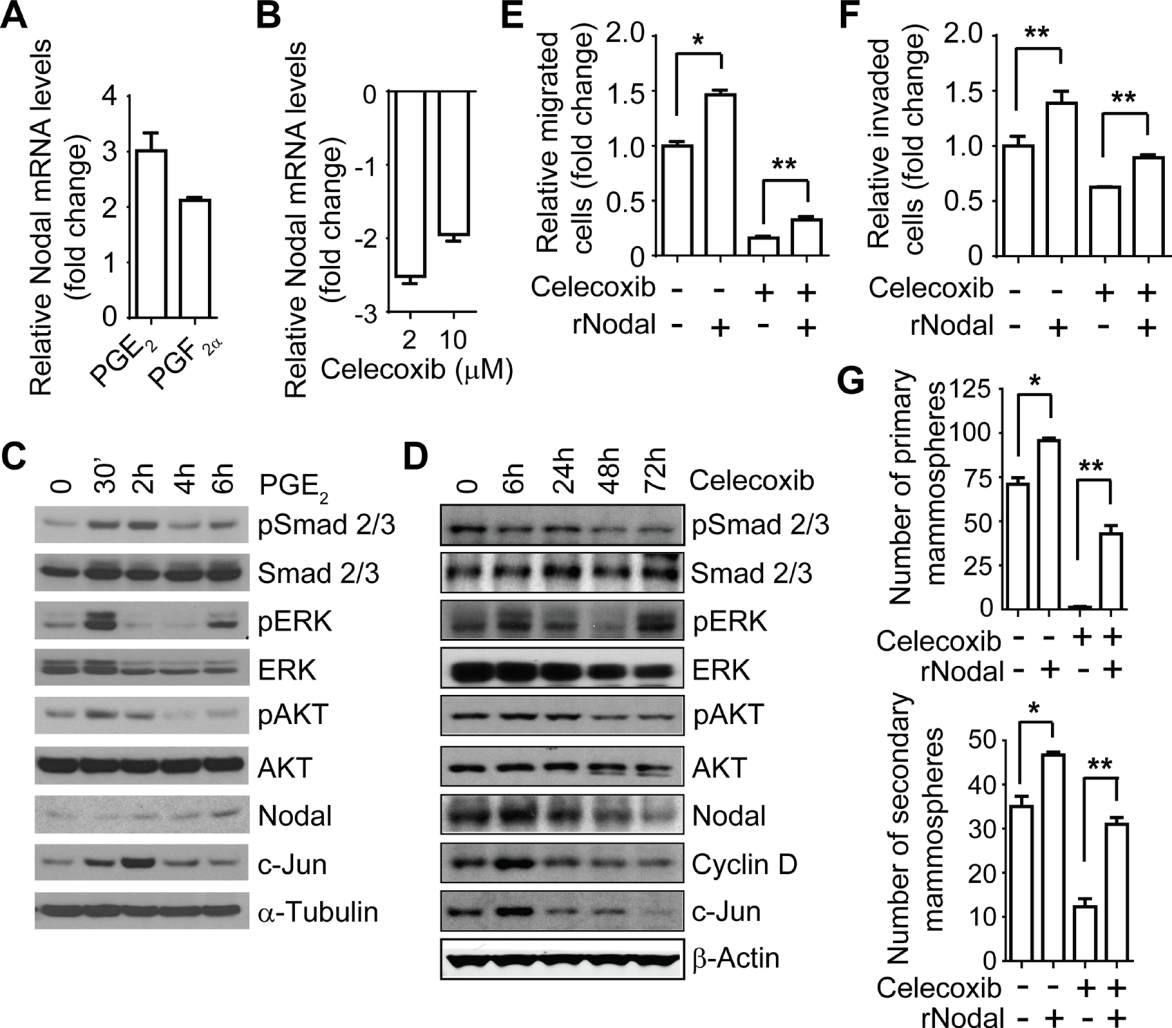
COX-2 pathway-regulated migration, invasion, and stem-like cell population are mediated by Nodal in IBC cells (**A**) and (**B**) The COX-2 pathway regulates Nodal mRNA in SUM149 cells. SUM149 cells were treated with 0.5 μM PGE_2_ or PGF_2α_ (A) or celecoxib at the indicated doses (B) for 48 hours under 3D culture conditions, and the expression level of Nodal was measured by real-time RT-PCR. (**C**) PGE_2_ stimulation activates the Nodal pathway in SUM149 cells. Serum-starved SUM149 cells were stimulated with 0.5 μM PGE_2_ for different time periods, and the expression of the indicated proteins was analyzed with Western blotting. (**D**) Celecoxib (10 μM) treatment reduces Nodal expression and inhibits the Nodal pathway in SUM149 cells. (**E**) and (**F**) Recombinant Nodal (rNodal) mitigates the celecoxib-induced inhibition of migration (E) and invasion (F) of SUM149 cells. Cells were treated with different combinations of celecoxib (25 μM) and rNodal (100 ng/mL) as indicated for 48 hours and then a transwell migration or Matrigel invasion assay was performed. *P < 0.001; **P < 0.05. (**G**) rNodal mitigates the celecoxib-induced inhibition of mammosphere formation of SUM149 cells. *P < 0.01; **P = 0.001. Experiments were independently repeated 3 times.

The findings of the above-described experiments, together with previous investigation of EGFR pathway in IBC tumorigenicity and metastasis [15], demonstrated that both the EGFR and COX-2 pathways regulate the EMT-like phenotype and the CSC marker-bearing population in IBC. To investigate the underlying mechanism, we then examined the effect of celecoxib on the expression of stem cell-related genes involved in EMT by using an EMT RT-PCR array. As shown in Supplementary Figure 5A, we selected 5 top target genes—SNAI1, SNAI2, Zeb2, Nodal, and ITGA5—that were downregulated by celecoxib treatment. We further validated the change in Nodal, a molecule in the TGFβ superfamily involved in regulating cell “stemness” [23–28]. Stimulation of SUM149 cells with PGE_2_ and PGF_2a_ increased Nodal expression at the mRNA level in SUM149 cells (Figure 5A), whereas celecoxib treatment decreased Nodal expression at the mRNA level in SUM149 cells (Figure 5B) and KPL-4 cells (Supplementary Figure 5B). We also observed the downregulation of Nodal expression in SUM149 xenograft tumors treated with celecoxib (250 and 500 ppm) (Figure 4H). These results suggest that Nodal may be a critical downstream molecule regulated by EGFR/COX-2 signaling in IBC.

To further confirm that Nodal is a potential mediator of the COX-2-regulated CSC phenotype, we examined the effect of PGE_2_ stimulation or celecoxib treatment on Nodal signaling. As shown in Figure 5C, PGE_2_ stimulation upregulated the phosphorylation of Smad2/3 and the downstream molecules ERK and AKT and upregulated the expression of Nodal, as well as COX-2 target gene cyclin D and Smad target gene c-Jun, in SUM149 cells, whereas treatment with celecoxib had the opposite effect in SUM149 (Figure 5D) and KPL-4 cells (Supplementary Figure 5C). Celecoxib treatment significantly reduced the expression of Nodal at the protein level in SUM149 (Figure 5D) and KPL-4 cells (Supplementary Figure 5C). Using another COX-2 inhibitor, CAY10404, we observed similar inhibitory effects on Nodal expression and signaling in SUM149 cells (Supplementary Figure 5D). These results confirmed that the COX-2 pathway regulates Nodal expression and signaling in IBC cells.

We further examined whether recombinant human Nodal (rNodal) treatment could rescue the inhibitory impact of celecoxib on IBC cells. As shown in Figure 5E and 5F, treatment of SUM149 cells with rNodal increased their ability to migrate and invade while mitigating celecoxib-induced inhibition of migration and invasion. rNodal also increased the formation of primary and secondary mammospheres in SUM149 cells (Figure 5G). Moreover, rNodal treatment mitigated the inhibitory effects of celecoxib on mammosphere formation of SUM149 cells (Figure 5G). These results suggest that Nodal is a potential key mediator of COX-2-regulated invasive capacity and the CSC population of IBC cells.

**Figure 6:**
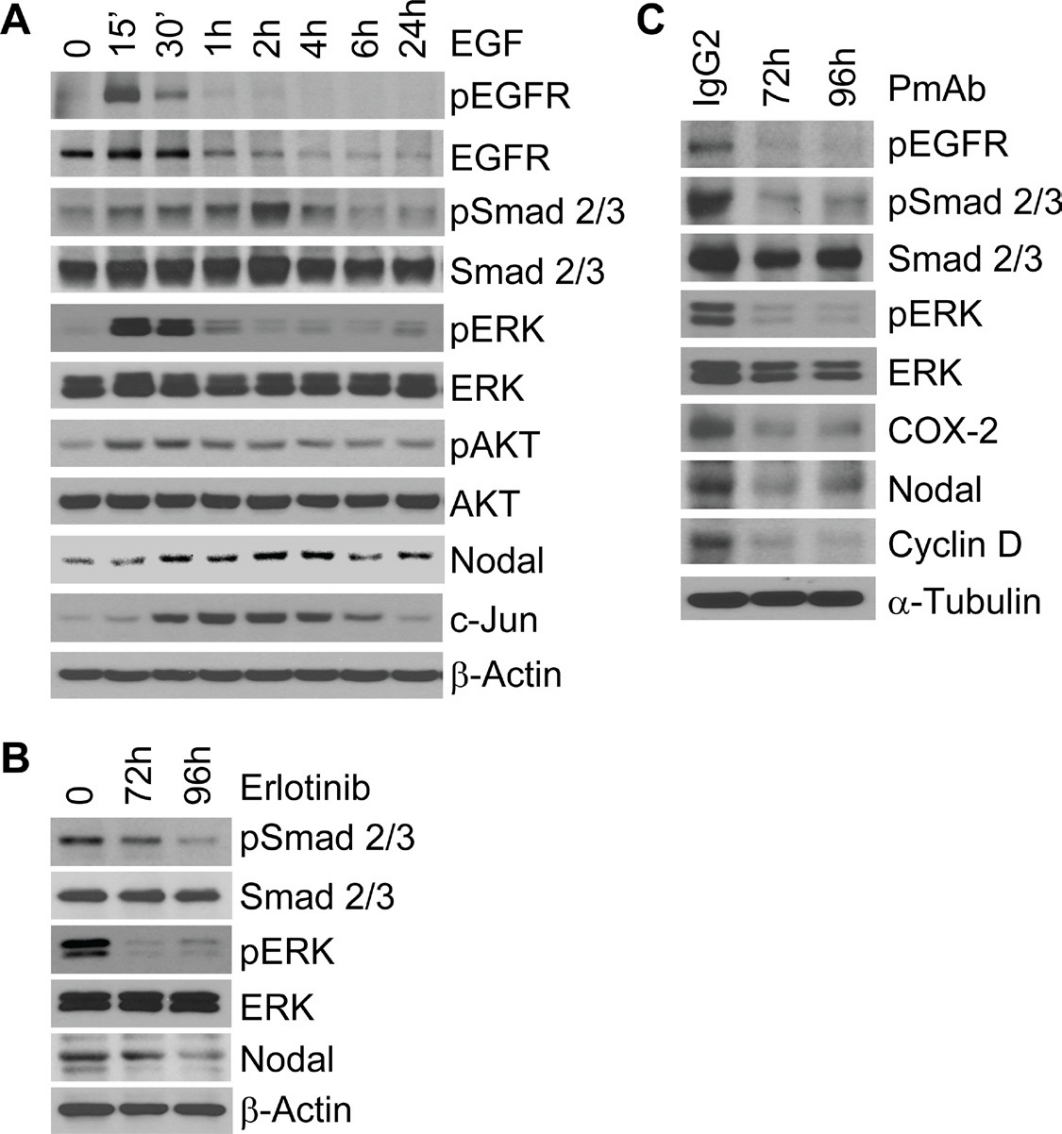
The EGFR pathway regulates Nodal signaling in IBC cells (**A**) EGF stimulation activates the Nodal pathway in SUM149 cells. Serum-starved SUM149 cells were stimulated with 50 ng/mL EGF for different time periods, and the expression of the indicated proteins was analyzed with Western blotting. (**B**) Erlotinib (2.5 μM) treatment reduces Nodal expression and inhibits the Nodal pathway in SUM149 cells. (**C**) PmAb (20 μg/mL) treatment reduces Nodal expression and inhibits the Nodal pathway in SUM149 cells. Experiments were independently repeated 3 times.

Next, we asked whether EGFR signaling regulates CSCs through Nodal. We stimulated SUM149 cells with EGF and found it upregulated the phosphorylation of Smad2/3 and downstream molecules ERK and AKT and the expression of Nodal and Smad target gene c-Jun (Figure 6A). In contrast, erlotinib treatment reduced the phosphorylation of Smad2/3 and ERK and the expression of Nodal in SUM149 mammospheres (Figure 6B). Similarly, inhibition of the EGFR pathway by PmAb treatment also reduced Nodal expression and inhibited Nodal signaling in SUM149 cells (Figure 6C). PmAb treatment also inhibited Nodal signaling in MDA-IBC3 cells (Supplementary Figure 5E). Using another EGFR tyrosine kinase inhibitor, gefitinib, we observed similar inhibitory effects on Nodal expression and signaling in SUM149 cells (Supplementary Figure 5F). These results suggest that EGFR regulates the activity of the Nodal pathway in IBC cells.

## DISCUSSION

In this study, we identified a novel EGFR/COX-2/Nodal axis that regulates IBC stem-like and EMT features. We have shown that 1) the EGFR pathway regulates the expression of COX-2, a key molecule in the inflammatory response whose expression correlates with worse outcome of IBC patients and whose inhibition reduces IBC cells’ invasion and tumor growth; 2) both the EGFR and COX-2 pathways contribute to the regulation of IBC stemness; and 3) Nodal, a molecule involved in the regulation of stem cell pluripotency, is a key component in EGFR/COX-2-mediated CSC regulation in IBC, and both the EGFR and COX-2 pathways regulate Nodal signaling. Our findings, for the first time, bring together several exciting avenues of research related to the distinct manifestation of IBC: evidence related to involvement of the EGFR signaling pathway, CSCs, and inflammation. Increasing evidence has suggested that CSCs contribute to acquired resistance to chemotherapy [29]; moreover, others have shown that the metastatic, aggressive behavior of IBC is mediated by a CSC component that displays ALDH enzymatic activity [11]. It is therefore intriguing to have found that the EGFR pathway promotes the cancer stem-like phenotype in IBC in the current study.

While there are clinicopathological inflammatory manifestations in IBC and the evidence of a physiologic inflammatory response [30], the molecular mechanism of how inflammation contributes to metastasis and progression of IBC remains elusive. Crosstalk between EGFR and COX-2 has been observed in other cancer types, but to our knowledge, the current study is the first to reveal the regulation of COX-2 by EGFR signaling in IBC and the positive correlation between EGFR and COX-2 expression in IBC patient samples. Moreover, we showed that COX-2 expression predicts worse overall survival of patients with IBC, COX-2 regulates self-renewal of CSCs and invasiveness of IBC cells, and COX-2 is required for EGFR regulation of CSC marker-expressing cells, highlighting the clinical implication of targeting COX-2 in IBC.

Transactivation of EGFR by COX-2-derived PGE_2_—thereby initiating a positive feedback loop between EGFR and COX-2 signaling—has been reported in some cancer types [31–33]. However, we did not observe the activation of EGFR signaling upon PGE_2_ stimulation in IBC SUM149 cells (data not shown). The lack of activation may be due to a different context or different experimental conditions, as one study showed that the ability of PGE_2_ to transactivate EGFR was rapid and depended on matrix metalloproteinase activity [32]. Our current data thus do not exclude the possibility that EGFR signaling can be transactivated by prostaglandins in IBC.

Our recent publications demonstrate that the tumor microenvironment is a critical driver of the IBC clinical phenotype and EGFR signaling plays an important role in mediating the crosstalk between IBC tumors and their microenvironment [34, 35]. Elucidating the mechanism of how EGFR regulates the crosstalk between IBC cells and the microenvironment will lead to the identification of other novel combination approaches for EGFR targeted therapy. Zelenay et al recently reported that tumor-derived COX activity is the key suppressor of type I IFN- and T cell-mediated tumor elimination and that COX-dependent immune evasion is critical for tumor growth in melanoma, colorectal, and breast cancer models [36]. Given that EGFR signaling regulates COX-2 in IBC and COX-2 contributes to aggressiveness of IBC, EGFR/COX-2 signaling may play a role in regulating the crosstalk between IBC cells and stroma; this warrants further investigation.

**Figure 7:**
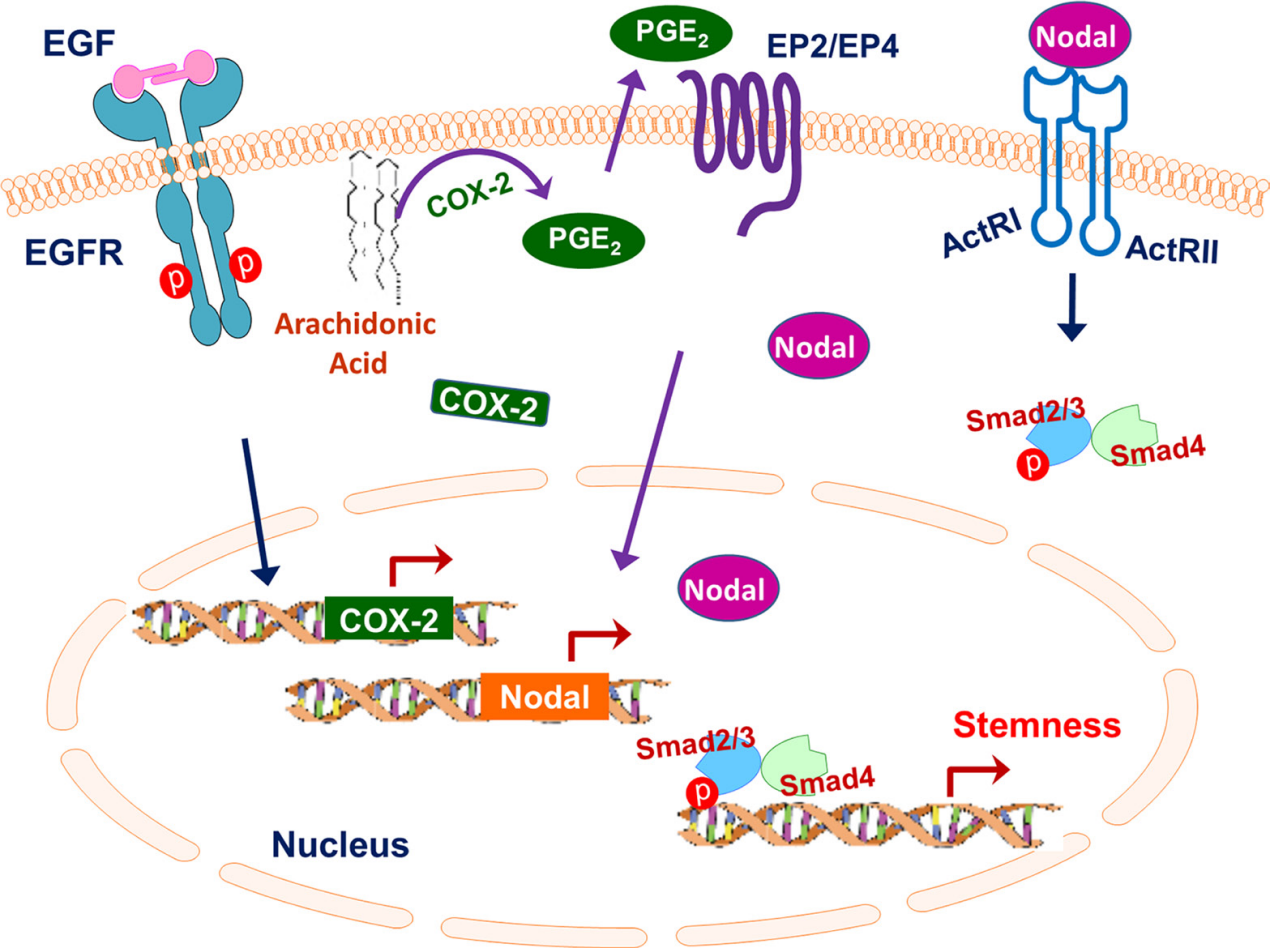
Schematic summarizing the role of the EGFR/COX-2/Nodal signaling axis in regulating IBC stemness EGFR signaling upregulates the expression of COX-2, which subsequently increases the production of prostaglandins such as PGE_2_ and PGF_2α_ in IBC cells; the activation of the COX-2 pathway upregulates the transcription of Nodal and activates Nodal signaling, which subsequently regulates IBC stemness.

Another novel finding of our study is the identification of Nodal as a downstream effector of EGFR/COX-2 activity in IBC. Nodal regulates the self-renewal of pancreatic CSCs [26, 27] and plays a role in sustaining tumor-initiating cell phenotypes and promoting invasiveness in breast cancer [24, 28, 37, 38]. Here, we demonstrated that EGFR and COX-2 regulate the expression and activity of Nodal signaling in IBC. Exogenous Nodal protein rescued the inhibitory effects of COX-2 inhibitor celecoxib on the IBC CSC population and invasiveness. Our data further indicated that COX-2 is required for EGFR regulation of CSCs because PGE_2_ rescued the inhibitory effect of EGFR depletion on mammosphere formation of SUM149 cells. On the basis of these results, we propose that EGFR signaling upregulates the expression of COX-2, which subsequently increases the production of prostaglandins such as PGE_2_ and PGF_2α_ in IBC cells; the activation of the COX-2 pathway upregulates the transcription of Nodal and activates Nodal signaling, which subsequently regulates IBC stemness (Figure 7).

Although our results highlight the clinical significance of targeting both the EGFR and COX-2 pathways in patients with IBC, we recognize that combination treatment of EGFR and COX-2 inhibitors in breast cancer has not been clinically successful. The identification of the EGFR/COX-2/Nodal axis as an IBC CSC regulator therefore provides a potential avenue of developing a combination approach that augments the efficacy of EGFR targeted therapy by targeting not only EGFR but also inflammation and CSC-related molecules in IBC, which might eventually lead to more impactful anti-EGFR clinical trials for patients with IBC. We are currently determining drug combinations targeting EGFR, COX-2, and Nodal signaling with synergistic effect in IBC cells and xenograft models. Although celecoxib is an effective chemopreventive agent against colon carcinogenesis, it has been reported that high doses of celecoxib can produce serious side effects, especially cardiac damage. Thus, we will consider other inhibitors of the COX-2 pathway with less toxicity in our future studies, such as inhibitors targeting COX-2 major metabolite PGE_2_ or its receptors EP2 and EP4.

In summary, we have identified an important signaling axis, EGFR/COX-2/Nodal, that promotes self-renewal of IBC CSCs and elucidated the role of the inflammatory process in regulating CSCs, invasiveness, and tumorigenesis of IBC. Considering that CSCs and inflammation have implications as major mechanisms of chemotherapy resistance, we expect our study to allow the development of EGFR targeted therapy with enhanced efficacy.

## MATERIALS AND METHODS

### Cell lines and reagents

The human IBC cell lines SUM149 and SUM190 were purchased from Asterand and were grown in Ham's F12 medium supplemented with 5% FBS, 5 μg/mL insulin, 1 μg/mL hydrocortisone, and 1% antibiotic-antimycotic. The human IBC cell lines KPL-4 [39] and FC-IBC-02 were kindly provided by Dr. Junichi Kurebayashi (Kawasaki Medical School, Japan) and Dr. Massimo Cristofanilli (Fox Chase Cancer Center), respectively, and both were maintained in Dulbecco's modified Eagle's medium/F12 medium supplemented with 10% FBS and 1% antibiotic-antimycotic. The human IBC cell line MDA-IBC3 was established by Dr. Wendy Woodward's laboratory in MD Anderson Cancer Center's Morgan Welch Inflammatory Breast Cancer Research Program and Clinic and were grown in Ham's F12 medium supplemented with 10% FBS, 5 μg/mL insulin, 1 μg/mL hydrocortisone, and 1% antibiotic-antimycotic. Non-IBC breast cancer cell lines BT-474, SK-BR-3, and MDA-MB-231, purchased from American Type Culture Collection, were grown in Dulbecco's modified Eagle's medium/F12 medium supplemented with 10% FBS. Non-IBC and IBC human breast cancer cell lines were validated using a short-tandem-repeat method based on a primer extension to detect single-base derivations by the Characterized Cell Line Core Facility at MD Anderson.

The following reagents were used: EGF (Sigma-Aldrich Chemical Co.), PGE_2_ and PGF_2a_ (Cayman Chemical), recombinant human Nodal (R&D Systems), PmAb (Amgen), erlotinib and celecoxib (Selleck Chemicals), dmPGE_2_ and CAY10404 (Cayman). An Aldefluor kit was purchased from Stemcell Technologies. The RT^2^ Profiler Human Epithelial to Mesenchymal Transition (EMT) PCR Array Kit was from SABiosciences. Details about the antibodies used are described in the Supplementary Materials and Methods.

### Generation of EGFR stable knockdown clones

Mission lentiviral transduction particles targeting EGFR were purchased from Sigma-Aldrich. The generation of EGFR stable knockdown clones in SUM149 cells was performed according to the manufacturer's instructions. The sequences of shEGFR are described in the Supplementary Materials and Methods.

### Mammosphere formation assay

Primary and secondary mammospheres of IBC cells were cultured in ultra-low-attachment 6-well plates (Corning Inc.) using MammoCult Basal Medium (human; Stemcell Technologies) supplemented with MammoCult Proliferation Supplements (human; Stemcell Technologies), 0.48 μg/mL hydrocortisone, and 4 μg/mL heparin for 7 days. For SUM149 cells, 20,000 cells/well or 10,000 cells/well were used for primary or secondary mammosphere formation, respectively, according to the manufacturer's instructions and previous report [40]. The following cell numbers were optimized and used for the KPL-4, MDA-IBC3, and FC-IBC-2 mammosphere formation assay: primary mammosphere formation: KPL-4, 5,000 cells/well; MDA-IBC3, 20,000 cells/well; FC-IBC-02, 20,000 cells/well; secondary mammosphere formation: KPL-4, 2,000 cells/well; MDA-IBC3, 10,000 cells/well; FC-IBC-02, 10,000 cells/well. Spheres were then stained with MTT (0.4 mg/mL), and the number of spheres bigger than 80 μm was counted with GelCount (Oxford Optronix).

### Flow cytometry assay

The ALDH activity and CD44^+^/CD24^−/low^ population of SUM149 EGFR knockdown clones or IBC cells treated as indicated in the figures were measured with flow cytometry. The details of these assays are described in the Supplementary Materials and Methods.

### Immunohistochemical staining and evaluation

Tissues from 44 patients with primary IBC who were treated at The University of Texas MD Anderson Cancer Center from September 1994 to August 2004 were included in this study. This study was approved by the MD Anderson Cancer Center Institutional Review Board. IHC staining of COX-2 was performed as described previously [41]. The IHC results were evaluated as described in detail in the Supplementary Materials and Methods.

### Three-dimensional (3D) culture, migration and invasion assays

IBC cells were treated with PGE_2_, PGF_2a_, or celecoxib for 48 hours and then subjected to three-dimensional culture, Boyden chamber migration assay, and Matrigel invasion assay as previously described [10].

### Prostaglandin extraction and analysis

Endogenous PGE_2_ and PGF_2α_ were extracted from IBC and non-IBC cells and SUM149 xenograft models, and prostaglandin levels were analyzed by using quantitative high-performance liquid chromatography tandem mass spectrometry (HPLC-MS/MS) according to the protocol of Yang et al. [42], as described in the Supplementary Materials and Methods.

### RT^2^ profiler human epithelial to mesenchymal transition (EMT) PCR array assay

SUM149 cells were treated with DMSO or 10 μM celecoxib for 48 hours, and then total DNA-free RNA was purified with an RNeasy Kit according to the manufacturer's instructions (Qiagen). cDNA was generated from 0.5 μg total RNA with the reverse transcriptase First Strand Kit and analyzed for stem cell-specific gene expression with the RT^2^ Profiler Human EMT PCR Array Kit (SABiosciences), as described in the Supplementary Materials and Methods.

### Xenograft studies

The animal experiment was approved by the Institutional Animal Care and Use Committee (IACUC protoco**l** 02-03-02134) of MD Anderson Cancer Center. A total volume of 0.15 mL of SUM149 cell suspension containing 2 × 10^6^ cells with 50% Matrigel was injected into the fourth inguinal mammary gland of 8-week-old female *nu*/*nu* mice. The mice were fed *ad libitum* with a regular diet for 3 weeks, at which time the tumors were well established. The mice were then randomly allocated to control diet or to one of two treatment diets (containing either 250 or 500 ppm of celecoxib) for another 5 weeks. Eight mice were included in each group. Tumor volume was measured weekly, and tumor growth inhibition was calculated as previously described [15].

### Quantitative RT-PCR

Total RNA was extracted and purified using an RNeasy mini kit (Qiagen, Inc.) according to the manufacturer's instructions. The quantitative RT-PCR reactions were performed using an iScript One-Step RT-PCR kit with SYBR Green (Bio-Rad). 7S rRNA mRNA was used as a normalization control. Primer sequences are described in the Supplementary Materials and Methods.

### Statistical analysis

Data are presented as mean ± SD. When two groups were compared, Student's *t*-test was used. Statistical analysis of the correlation between COX-2 expression and IBC patient survival was performed using log-rank test. *P* < 0.05 was considered statistically significant.

## SUPPLEMENTARY TABLE AND FIGURES



## References

[1] Costa R, Santa-Maria CA, Rossi G, Carneiro BA, Chae YK, Gradishar WJ, Giles FJ, Cristofanilli M (2017). Developmental therapeutics for inflammatory breast cancer: Biology and translational directions. Oncotarget.

[2] Cristofanilli M, Buzdar AU, Hortobagyi GN (2003). Update on the management of inflammatory breast cancer. Oncologist.

[3] Charafe-Jauffret E, Tarpin C, Viens P, Bertucci F (2008). Defining the molecular biology of inflammatory breast cancer. Seminars in oncology.

[4] Jaiyesimi IA, Buzdar AU, Hortobagyi G (1992). Inflammatory breast cancer: a review. J Clin Oncol.

[5] Levine PH, Steinhorn SC, Ries LG, Aron JL (1985). Inflammatory breast cancer: the experience of the surveillance, epidemiology, and end results (SEER) program. J Natl Cancer Inst.

[6] van Golen KL, Davies S, Wu ZF, Wang Y, Bucana CD, Root H, Chandrasekharappa S, Strawderman M, Ethier SP, Merajver SD (1999). A novel putative low-affinity insulin-like growth factor-binding protein, LIBC (lost in inflammatory breast cancer), and RhoC GTPase correlate with the inflammatory breast cancer phenotype. Clin Cancer Res.

[7] Alpaugh ML, Tomlinson JS, Ye Y, Barsky SH (2002). Relationship of sialyl-Lewis(x/a) underexpression and E-cadherin overexpression in the lymphovascular embolus of inflammatory breast carcinoma. Am J Pathol.

[8] Van der Auwera I, Van Laere SJ, Van den Eynden GG, Benoy I, van Dam P, Colpaert CG, Fox SB, Turley H, Harris AL, Van Marck EA, Vermeulen PB, Dirix LY (2004). Increased angiogenesis and lymphangiogenesis in inflammatory versus noninflammatory breast cancer by real-time reverse transcriptase-PCR gene expression quantification. Clin Cancer Res.

[9] Silvera D, Arju R, Darvishian F, Levine PH, Zolfaghari L, Goldberg J, Hochman T, Formenti SC, Schneider RJ (2009). Essential role for eIF4GI overexpression in the pathogenesis of inflammatory breast cancer. Nat Cell Biol.

[10] Wang X, Saso H, Iwamoto T, Xia W, Gong Y, Pusztai L, Woodward WA, Reuben JM, Warner SL, Bearss DJ, Hortobagyi GN, Hung MC, Ueno NT (2013). TIG1 promotes the development and progression of inflammatory breast cancer through activation of Axl kinase. Cancer Res.

[11] Charafe-Jauffret E, Ginestier C, Iovino F, Tarpin C, Diebel M, Esterni B, Houvenaeghel G, Extra JM, Bertucci F, Jacquemier J, Xerri L, Dontu G, Stassi G (2010). Aldehyde dehydrogenase 1-positive cancer stem cells mediate metastasis and poor clinical outcome in inflammatory breast cancer. Clin Cancer Res.

[12] Cabioglu N, Gong Y, Islam R, Broglio K, Sneige N, Sahin A, Gonzalez-Angulo A, Morandi P, Bucana C, Hortobagyi G, Cristofanilli M (2007). Expression of growth factor and chemokine receptors: new insights in the biology of inflammatory breast cancer. Ann Oncol.

[13] Corkery B, Crown J, Clynes M, O'Donovan N (2009). Epidermal growth factor receptor as a potential therapeutic target in triple-negative breast cancer. Ann Oncol.

[14] Matsuda N, Lim B, Wang X, Ueno NT (2017). Early clinical development of epidermal growth factor receptor targeted therapy in breast cancer. Expert opinion on investigational drugs.

[15] Zhang D, LaFortune TA, Krishnamurthy S, Esteva FJ, Cristofanilli M, Liu P, Lucci A, Singh B, Hung MC, Hortobagyi GN, Ueno NT (2009). Epidermal growth factor receptor tyrosine kinase inhibitor reverses mesenchymal to epithelial phenotype and inhibits metastasis in inflammatory breast cancer. Clin Cancer Res.

[16] Matsuda N, Alvarez RH, Krishnamurthy S, Willey JS, Wang X, Lim B, Parker CA, Marx A, Babiera G, Booser DJ, Murray JL, Arun B, Brewster AM (2015). Phase II study of panitumumab, nab-paclitaxel, and carboplatin followed by FEC neoadjuvant chemotherapy for patients with primary HER-2 negative inflammatory breast cancer. Journal of Clinical Oncology.

[17] Balkwill F, Coussens LM (2004). Cancer: an inflammatory link. Nature.

[18] Hugo HJ, Saunders C, Ramsay RG, Thompson EW (2015). New Insights on COX-2 in Chronic Inflammation Driving Breast Cancer Growth and Metastasis. J Mammary Gland Biol Neoplasia.

[19] Kim HS, Moon HG, Han W, Yom CK, Kim WH, Kim JH, Noh DY (2012). COX2 overexpression is a prognostic marker for Stage III breast cancer. Breast cancer research and treatment.

[20] Wang D, Dubois RN (2010). The role of COX-2 in intestinal inflammation and colorectal cancer. Oncogene.

[21] Esbona K, Inman D, Saha S, Jeffery J, Schedin P, Wilke L, Keely P (2016). COX-2 modulates mammary tumor progression in response to collagen density. Breast Cancer Res.

[22] Harris RE (2009). Cyclooxygenase-2 (cox-2) blockade in the chemoprevention of cancers of the colon, breast, prostate, and lung. Inflammopharmacology.

[23] Bodenstine TM, Chandler GS, Seftor RE, Seftor EA, Hendrix MJ (2016). Plasticity underlies tumor progression: role of Nodal signaling. Cancer metastasis reviews.

[24] Gong W, Sun B, Sun H, Zhao X, Zhang D, Liu T, Zhao N, Gu Q, Dong X, Liu F (2017). Nodal signaling activates the Smad2/3 pathway to regulate stem cell-like properties in breast cancer cells. American journal of cancer research.

[25] Gong Y, Guo Y, Hai Y, Yang H, Liu Y, Yang S, Zhang Z, Ma M, Liu L, Li Z, He Z (2014). Nodal promotes the self-renewal of human colon cancer stem cells via an autocrine manner through Smad2/3 signaling pathway. BioMed research international.

[26] Lonardo E, Frias-Aldeguer J, Hermann PC, Heeschen C (2012). Pancreatic stellate cells form a niche for cancer stem cells and promote their self-renewal and invasiveness. Cell cycle.

[27] Lonardo E, Hermann PC, Mueller MT, Huber S, Balic A, Miranda-Lorenzo I, Zagorac S, Alcala S, Rodriguez-Arabaolaza I, Ramirez JC, Torres-Ruiz R, Garcia E, Hidalgo M (2011). Nodal/Activin signaling drives self-renewal and tumorigenicity of pancreatic cancer stem cells and provides a target for combined drug therapy. Cell stem cell.

[28] Quail DF, Zhang G, Findlay SD, Hess DA, Postovit LM (2014). Nodal promotes invasive phenotypes via a mitogen-activated protein kinase-dependent pathway. Oncogene.

[29] Baguley BC (2010). Multiple drug resistance mechanisms in cancer. Molecular biotechnology.

[30] Dawood S, Merajver SD, Viens P, Vermeulen PB, Swain SM, Buchholz TA, Dirix LY, Levine PH, Lucci A, Krishnamurthy S, Robertson FM, Woodward WA, Yang WT (2011). International expert panel on inflammatory breast cancer: consensus statement for standardized diagnosis and treatment. Annals of oncology.

[31] Buchanan FG, Wang D, Bargiacchi F, DuBois RN (2003). Prostaglandin E2 regulates cell migration via the intracellular activation of the epidermal growth factor receptor. The Journal of biological chemistry.

[32] Pai R, Soreghan B, Szabo IL, Pavelka M, Baatar D, Tarnawski AS (2002). Prostaglandin E2 transactivates EGF receptor: a novel mechanism for promoting colon cancer growth and gastrointestinal hypertrophy. Nature medicine.

[33] Shao J, Lee SB, Guo H, Evers BM, Sheng H (2003). Prostaglandin E2 stimulates the growth of colon cancer cells via induction of amphiregulin. Cancer Res.

[34] Lacerda L, Debeb BG, Smith D, Larson R, Solley T, Xu W, Krishnamurthy S, Gong Y, Levy LB, Buchholz T, Ueno NT, Klopp A, Woodward WA (2015). Mesenchymal stem cells mediate the clinical phenotype of inflammatory breast cancer in a preclinical model. Breast Cancer Res.

[35] Wolfe AR, Trenton NJ, Debeb BG, Larson R, Ruffell B, Chu K, Hittelman W, Diehl M, Reuben JM, Ueno NT, Woodward WA (2016). Mesenchymal stem cells and macrophages interact through IL-6 to promote inflammatory breast cancer in pre-clinical models. Oncotarget.

[36] Zelenay S, van der Veen AG, Bottcher JP, Snelgrove KJ, Rogers N, Acton SE, Chakravarty P, Girotti MR, Marais R, Quezada SA, Sahai E, Reis e Sousa C (2015). Cyclooxygenase-Dependent Tumor Growth through Evasion of Immunity. Cell.

[37] Bodenstine TM, Chandler GS, Reed DW, Margaryan NV, Gilgur A, Atkinson J, Ahmed N, Hyser M, Seftor EA, Strizzi L, Hendrix MJ (2016). Nodal expression in triple-negative breast cancer: Cellular effects of its inhibition following doxorubicin treatment. Cell cycle.

[38] Meyer MJ, Fleming JM, Ali MA, Pesesky MW, Ginsburg E, Vonderhaar BK (2009). Dynamic regulation of CD24 and the invasive, CD44posCD24neg phenotype in breast cancer cell lines. Breast Cancer Res.

[39] Kurebayashi J, Otsuki T, Tang CK, Kurosumi M, Yamamoto S, Tanaka K, Mochizuki M, Nakamura H, Sonoo H (1999). Isolation and characterization of a new human breast cancer cell line, KPL-4, expressing the Erb B family receptors and interleukin-6. British journal of cancer.

[40] Klopp AH, Lacerda L, Gupta A, Debeb BG, Solley T, Li L, Spaeth E, Xu W, Zhang X, Lewis MT, Reuben JM, Krishnamurthy S, Ferrari M (2010). Mesenchymal stem cells promote mammosphere formation and decrease E-cadherin in normal and malignant breast cells. PloS one.

[41] Sicking I, Rommens K, Battista MJ, Bohm D, Gebhard S, Lebrecht A, Cotarelo C, Hoffmann G, Hengstler JG, Schmidt M (2014). Prognostic influence of cyclooxygenase-2 protein and mRNA expression in node-negative breast cancer patients. BMC cancer.

[42] Yang P, Felix E, Madden T, Fischer SM, Newman RA (2002). Quantitative high-performance liquid chromatography/electrospray ionization tandem mass spectrometric analysis of 2- and 3-series prostaglandins in cultured tumor cells. Analytical Biochemistry.

