# Supplementary material for: EGFR signaling promotes inflammation and cancer stem-like activity in inflammatory breast cancer
Source: Oncotarget. 2017 Jul 4;8(40):67904–17. doi: 10.18632/oncotarget.18958 (PMC5620223; doi:10.18632/oncotarget.18958)
Supplement: Supplementary file 1 [file oncotarget-08-67904-s001.pdf]

EGFR signaling promotes inflammation and cancer stem-like activity in inflammatory breast cancer

Supplementary Materials

MATERIALS AND METHODS

Generation of EGFR stable knockdown clones

To generate EGFR stable knockdown clones in SUM149 cells, two Mission ShRNA Transduction Particles targeting human EGFR (Sigma-Aldrich) were used according to the manufacturer's guidelines: shEGFR-1 (CCGGAGAATGTGGAATACCTAAGGCT CGAGCCTTAGGTATTCCACATTCTCTTTTTG) and shEGFR-3 (CCGGGCCACAAAGCAGTGAATTTATC TCGAGATAAATTCACTGCTTTGTGGCTTTTTG). Mission Non-Target shRNA Control Transduction Particles (SHC002V) were used as a control. Stable control (shCtrl) and EGFR knockdown clones (shEGFR-1 and shEGFR-3) were selected and maintained with puromycin.

Immunoblotting

Cells were lysed in RIPA buffer (50 mmol/L Tris-HCl [pH 7.5], 150 mmol/L NaCl, 1% NP40, 0.5% sodium deoxycholate, 0.1% SDS) supplemented with 10 µL/mL phosphatase inhibitor cocktail and 10 µL/mL protease inhibitor cocktail. SDS-PAGE and immunoblotting were carried out according to standard procedures. The following primary antibodies were used: anti-EGFR (Santa Cruz Biotechnology), anti-COX-2 (Cayman Chemical), anti-E-cadherin or -fibronectin (BD Transduction), anti-phospho-EGFR (Y1086), -pERK, -ERK, -pAKT, -AKT, -c-Jun, -vimentin, or -N-cadherin (Cell Signaling), anti-pSmad 2/3 or -Smad 2/3 (Millipore), anti-cyclin D (Thermo Scientific), anti-Nodal (Abcam), and anti-β-actin or -α-tubulin (Sigma-Aldrich Chemical Co.).

Flow cytometry assay

The ALDH enzymatic activity was measured by using an Aldefluor kit (Stemcell Technologies, Vancouver, Canada) according to the manufacturer's instructions. Briefly, cells were resuspended in Aldefluor assay buffer containing ALDH substrate. Cells treated with N,N-diethylaminobenzaldehyde (DEAB), an inhibitor of ALDH activity, were used as a control. Cells were then incubated at 37°C for 30 minutes, washed with Aldefluor assay buffer, and stained with 1 µg/mL propidium iodide (Sigma) to discriminate viable cells from dead cells before detection in the green fluorescence channel on the flow cytometer. For the $CD44^{+}/CD24^{-/low}$ subpopulation assay, combinations of fluorochrome-conjugated monoclonal antibodies against human CD44 (fluorescein isothiocyanate, BD Biosciences) and CD24 (phycoerythrin, BD Biosciences) or their respective isotype controls (BD Biosciences) were added to the cell suspension at concentrations recommended by the manufacturer and incubated at room temperature in the dark for 30 minutes. The labeled cells were washed and resuspended in FACS buffer and then analyzed on a FACSVantage flow cytometry system (BD Biosciences).

Immunohistochemical staining and evaluation

Tissues from patients with primary IBC who were treated at The University of Texas MD Anderson Cancer Center between September 1994 and August 2004 were used in this study. This study was approved by the MD Anderson Cancer Center Institutional Review Board. Formalin-fixed paraffin-embedded tissues were used to construct a tissue microarray (TMA) as described previously [1]. IHC staining for COX-2 on TMA slides was performed as described previously [2]. Positive COX-2 status was defined as cytoplasmic staining in at least 10% of tumor cells. The COX-2 protein level was scored using an H-scoring system obtained by multiplying the staining intensity (graded as 0, negative; +, weak; ++, moderate; and +++, strong) by the percentage of epithelial tumor cells with positive staining for the respective proteins (1–100%). The cut-off value of 75 was used to define low/high expression of COX-2.

Prostaglandin extraction and analysis

Briefly, cell pellets were suspended in 0.5 mL of PBS, 40 µL of 1 N citric acid, and 5 µL of 10% (w/v) butylated hydroxytoluene. Prostaglandins were extracted by liquid-liquid extraction using 2 mL of 1/1 ethyl acetate/hexanes (v/v), three times. The organic layers were separated, pooled, and evaporated to dryness under nitrogen. The samples were reconstituted in 100 µL of 50/50 methanol/0.1% acetic acid (v/v). Prostaglandins were detected using an Agilent 6460 triple quadrupole mass spectrometer (Agilent Technologies) equipped with an Agilent HP 1200 binary HPLC pump. PGE_2 and $PGF_{2\alpha}$ were separated using a 2 × 100-mm Kinetex 3 µm

C18 analytical column (Phenomenex). Mobile phase A consisted of 0.1% formic acid, and mobile phase B was acetonitrile with 0.1% formic acid. Compounds were eluted with a gradient starting at 20% B and ramped to 90% B over 14 minutes. The column temperature was maintained at 40°C, and samples were kept at 4°C during the analysis.

Quantitative RT-PCR

The relative quantitation value for each target gene compared to the calibrator for that target was expressed as $2^{-(Ct-Cc)}$ (Ct and Cc are the mean threshold cycle differences after normalization to 7S rRNA). The sequences of the primers used in this study were as follows: 7S: forward 5′-ATCGGGTGTCCGCACTAAGTT-3′; reverse 5′-CAGCACGGGAGTTTTGACCT-3′; E-cadherin: forward 5′-AGTGCCAACTGGACCATTCA-3′, reverse 5′-TCTTTGACCACCGCTCTCCT-3′; N-cadherin: forward 5′-ACTCGCAGACGCTCACACGC-3′, reverse 5′-GCGGGACTCGCACCAGGAGT-3′; fibronectin: forward 5′-CCATCACTGTGTATGCTGTC-3′, reverse 5′-TGGTTTGTCAATTTCTGTTCGG-3′; Snail: forward 5′-TCCAGGCTCGAAAGGCCTTCAAC-3′, reverse 5′-GCAGCGTGTGGCTTCGGATGT-3′; Slug: forward 5′-GGGTGACTTCAGAGGCGCCG-3′, reverse 5′-GGCGGTCCCTACAGCATCGC-3′; vimentin: forward 5′-CAAGGGCCAAGGCAAGTCGCG-3′, reverse 5′-ACGCGGGCTTTGTCGTTGGTTA-3′; and Nodal: forward 5′-AGCATGGTTTTGGAGGTGAC-3′, reverse 5′-CCTGCGAGAGGTTGGAGTAG-3′.

RT² profiler human EMT PCR array assay

Quantitative reverse transcriptase–polymerase chain reaction (qRT-PCR) was performed on a Bio-Rad CFX-96 Real-Time PCR Detection System (Bio-Rad Laboratories, Inc.). Data were collected and analyzed using the threshold-cycle (Ct) relative quantification method. Experiments were performed in triplicate. For each gene, fold-change was calculated as the difference in gene expression between SUM149 cells treated with DMSO and SUM149 cells treated with celecoxib. A positive value indicated gene upregulation, and a negative value indicated gene downregulation. The *P* values were calculated based on a Student's *t*-test of the replicate 2^(- delta Ct) values for each gene in the control group and treatment groups; *P* values less than 0.05 were considered significant.

REFERENCES

1. Gong Y, Huo L, Liu P, Sneige N, Sun X, Ueno NT, Lucci A, Buchholz TA, Valero V, Cristofanilli M. Polycomb group protein EZH2 is frequently expressed in inflammatory breast cancer and is predictive of worse clinical outcome. Cancer. 2011; 117:5476–5484.
2. Sicking I, Rommens K, Battista MJ, Bohm D, Gebhard S, Lebrecht A, Cotarelo C, Hoffmann G, Hengstler JG, Schmidt M. Prognostic influence of cyclooxygenase-2 protein and mRNA expression in node-negative breast cancer patients. BMC cancer. 2014; 14:952.

Supplementary Table 1: Relation between COX-2 expression status and clinicopathologic factors of 44 patients with IBC

	COX-2 Expression Status		
**Prognostic Factor**	**Low (*n* = 20)**	**High (*n* = 24)**	***P***
**Age at diagnosis, y**			
Medium (range)	45 (32–69)	47.5 (23–75)	0.56
**Race/ethnicity**			
Hispanic	2	2	0.99
White	17	21	
Others	1	1	
**Histologic type**			
Ductal	15	22	0.29
Lobular	2	1	
Others	3	1	
**Nuclear grade**			
II	6	3	0.026
III	14	21	
**Lymphovascular invasion**			
No	3	2	0.82
Yes	13	21	
Unknown	4	1	
**ER expression**			
Negative	9	15	0.29
Positive	11	9	
**PR expression**			
Negative	11	14	0.71
Positive	7	10	
**HER2 expression**			
Negative	12	17	0.59
Positive	8	7	

Abbreviations: ER, estrogen receptor; PR, progesterone receptor; HER2, human epidermal growth factor receptor 2. *P* values were calculated using the Pearson chi-square test.

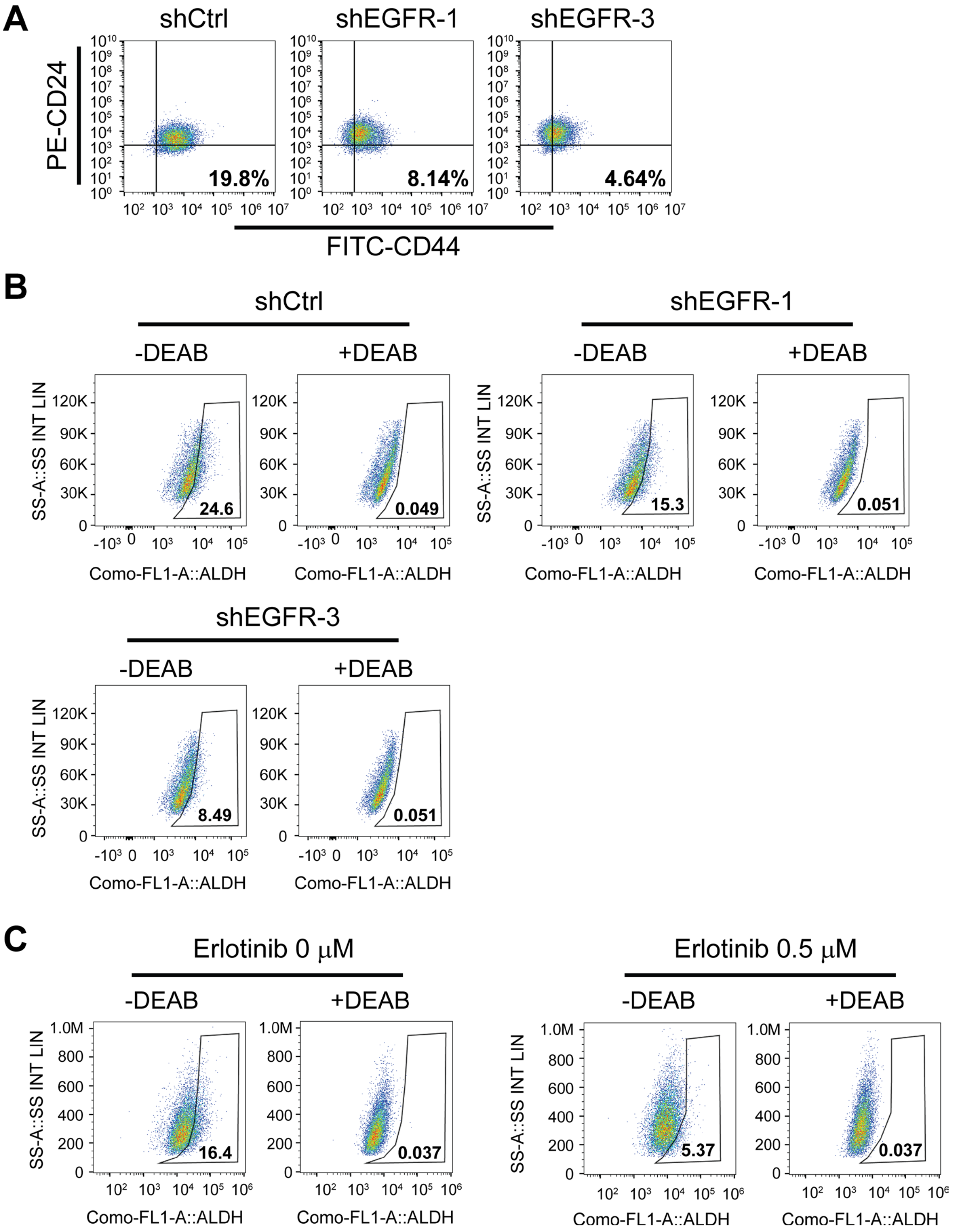

Supplementary Figure 1: (**A**) and (**B**). Gating schemes for the CD44+/CD24-/low population (A) and ALDH activity (B) in the SUM149 shCtrl clone and shEGFR clones 1 and 3. (**C**) Gating schemes for ALDH activity in SUM149 cells treated with erlotinib.

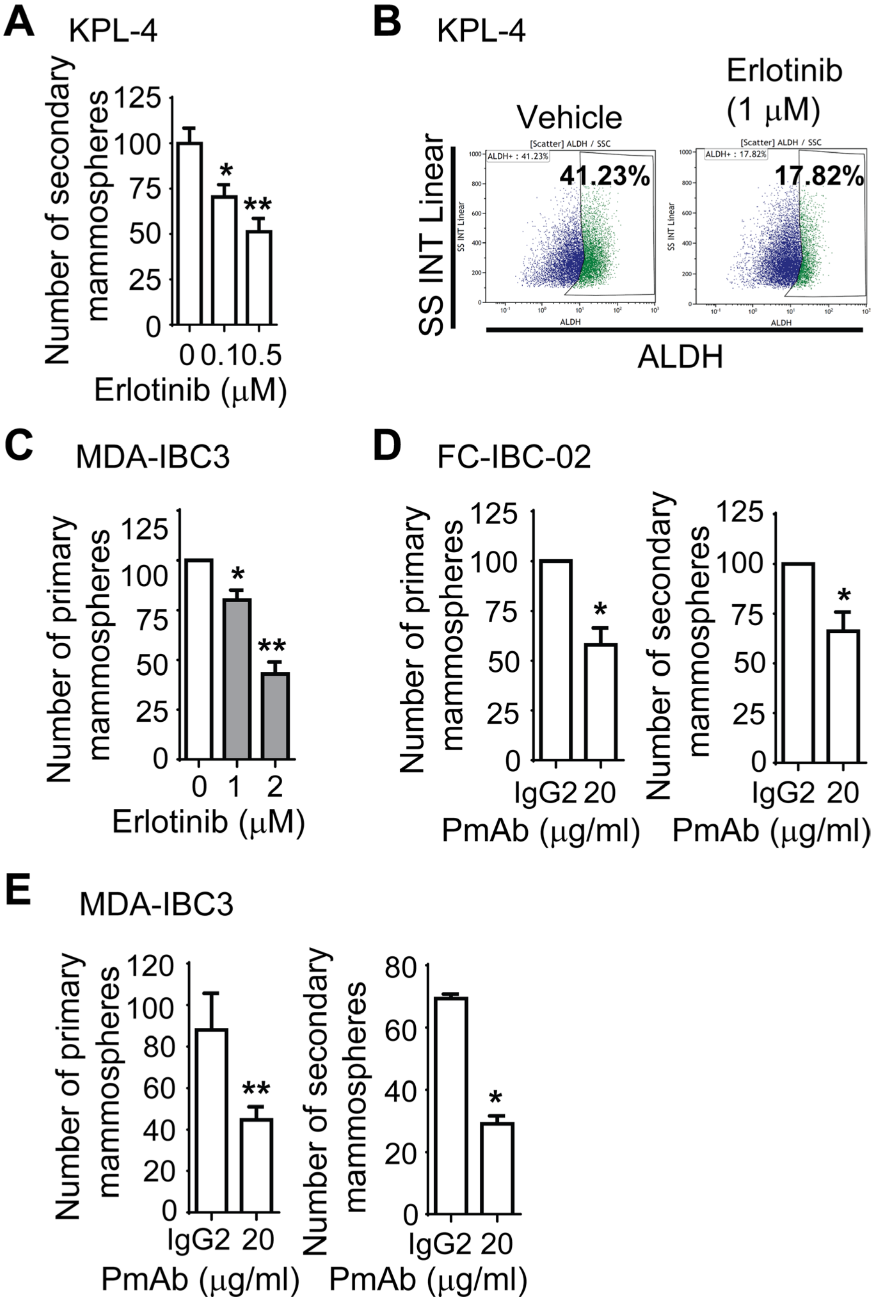

Supplementary Figure 2: The EGFR pathway regulates the IBC cell population that expresses CSC markers. (**A**) Erlotinib treatment at the indicated doses decreases mammosphere formation of KPL-4 cells. *P < 0.01; **P < 0.005. (**B**) Erlotinib treatment decreases ALDH activity of KPL-4 cells. Cells were treated with 1 μM erlotinib for 48 hours and then subjected to flow cytometry analysis. (**C**) Erlotinib treatment at the indicated doses decreases mammosphere formation of MDA-IBC3 cells. *P < 0.005; **P < 0.001. (**D**) and (**E**) PmAb treatment decreases the primary (left) and secondary (right) mammosphere formation of FC-IBC-02 (D) and MDA-IBC3 (E) cells. Mammosphere formation with or without PmAb (20 μg/mL) was measured. *P < 0.005; **P < 0.05. Experiments were independently repeated 3 times.

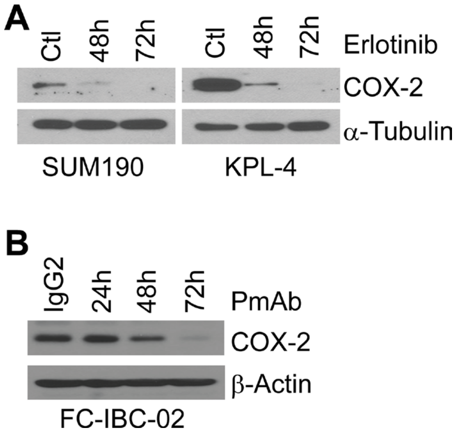

Supplementary Figure 3: The EGFR pathway regulates COX-2 expression in IBC cells. (**A**) Erlotinib treatment reduces the expression of COX-2 in SUM190 (left panel) and KPL-4 (right panel) cells. Cells were treated with erlotinib (1 μM) for different time periods, and the expression of COX-2 was analyzed with Western blotting. (**B**) PmAb treatment reduces the expression of COX-2 in FC-IBC-02 cells. Cells were treated with PmAb (100 μg/mL) for different time periods, and the expression of COX-2 was analyzed with Western blotting. Experiments were independently repeated 3 times.

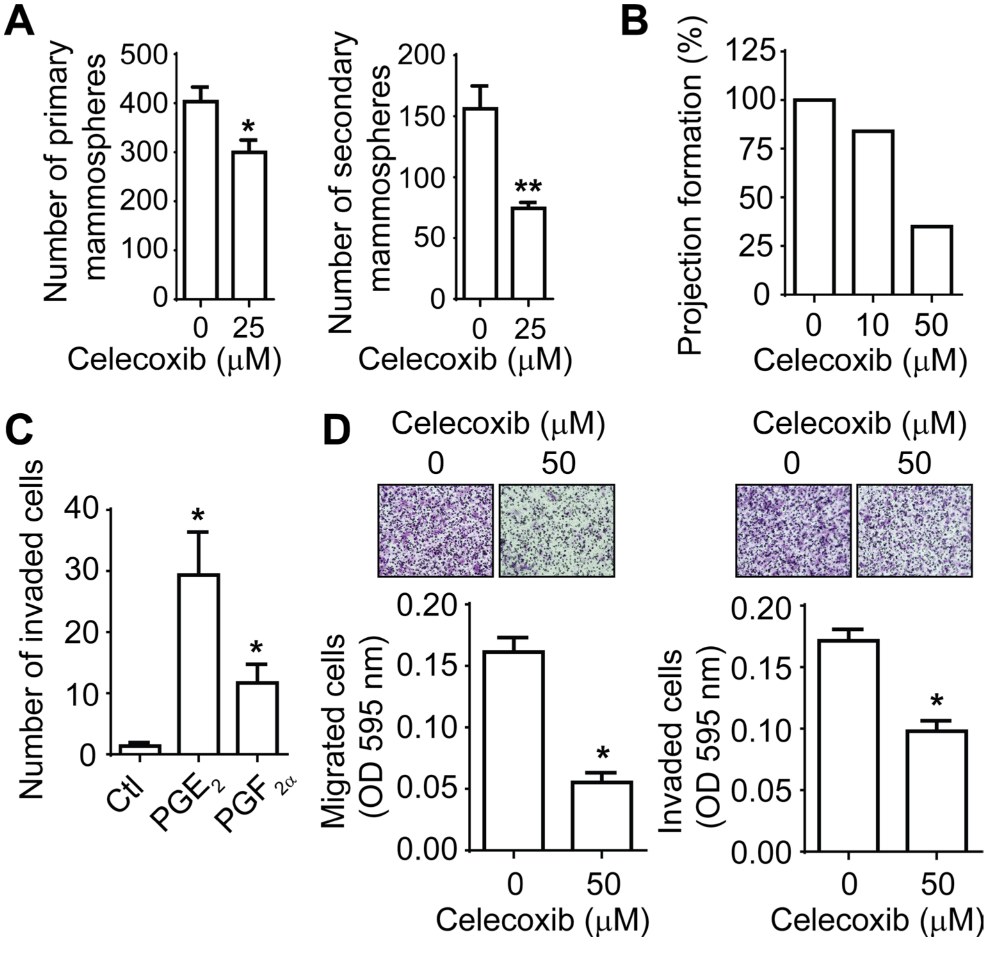

Supplementary Figure 4: The COX-2 pathway regulates invasiveness of IBC cells *in vitro*. (**A**) Celecoxib treatment decreases mammosphere formation of KPL-4 cells. Primary and secondary mammosphere formation of KPL-4 cells treated with celecoxib at the indicated dose was measured. *$P < 0.05$; **$P < 0.005$. (**B**) Celecoxib treatment reduces projection formation of KPL-4 cells. Cells were plated in Matrigel culture with or without celecoxib for 48 hours. Projections were quantitated by S.CORE analysis. (**C**) PGE_2 and $PGF_{2\alpha}$ treatment increases the invasion of KPL-4 cells. Cells were treated with 0.5 μM PGE_2 or $PGF_{2\alpha}$ for 48 hours and then subjected to Matrigel invasion assay. *$P < 0.005$. (**D**) Celecoxib treatment decreases the migration (left panel) and invasion (right panel) of KPL-4 cells. Cells were treated with DMSO as a control or 50 μM celecoxib for 24 hours, and then another assay for 6-hour transwell migration or 24-hour invasion through Matrigel was performed. *$P < 0.01$. Experiments were independently repeated 3 times.

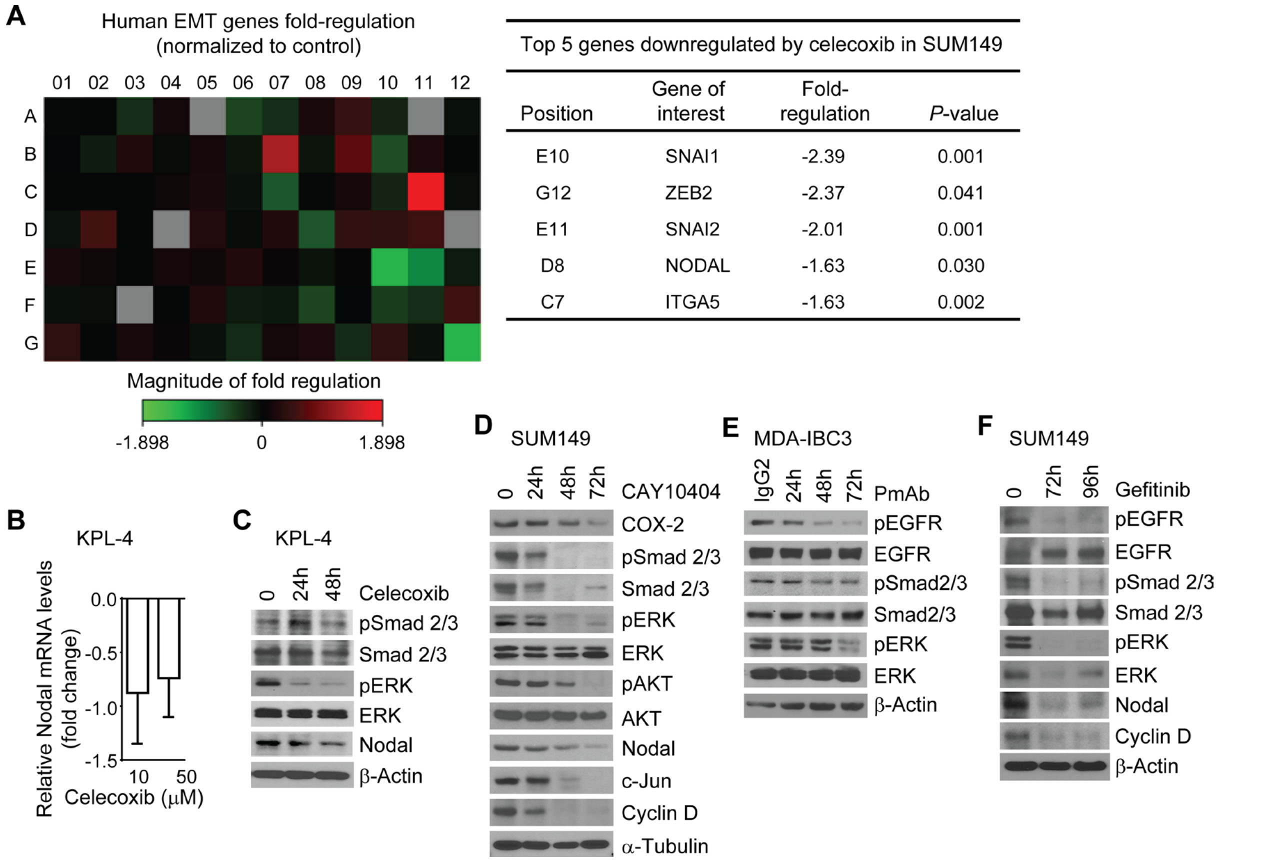

Supplementary Figure 5: The EGFR and COX-2 pathways regulate Nodal signaling in IBC cells. (**A**) Nodal is one of the top genes downregulated by celecoxib. Candidate genes involved in COX-2-regulated EMT phenotype and CSCs were identified by comparing the changes in stem cell-related genes involved in EMT after celecoxib treatment using an EMT RT-PCR array. Left panel: Heat map of array. Right panel: Top 5 genes downregulated by celecoxib treatment in SUM149 cells. (**B**) The COX-2 pathway regulates Nodal mRNA in KPL-4 cells. Cells were treated with celecoxib at indicated doses for 48 hours under 3D culture conditions, and the expression level of Nodal was measured by real-time RT-PCR. (**C**) Celecoxib treatment reduces Nodal expression and inhibits the Nodal pathway in KPL-4 cells. Cells were treated with 25 µM celecoxib for different time periods, and the expression of the indicated proteins was analyzed with Western blotting. (**D**) CAY10404 treatment reduces Nodal expression and inhibits the Nodal pathway in SUM149 cells. Cells were treated with 5 µM CAY10404 for different time periods, and protein expression was analyzed with Western blotting. (**E**) PmAb treatment inhibits the Nodal pathway in MDA-IBC3 cells. Cells were treated with 100 µg/mL PmAb for different time periods, and protein expression was analyzed with Western blotting. (**F**) Gefitinib treatment reduces Nodal expression and inhibits the Nodal pathway in MDA-IBC3 cells. Cells were treated with 2 µM gefitinib for different time periods, and protein expression was analyzed with Western blotting.
